# Epidemiological and clinical features of rotavirus among children younger than 5 years of age hospitalized with acute gastroenteritis in Northern Italy

**DOI:** 10.1186/1471-2334-10-218

**Published:** 2010-07-22

**Authors:** Gianvincenzo Zuccotti, Fabio Meneghin, Dario Dilillo, Luisa Romanò, Roberta Bottone, Cecilia Mantegazza, Roberto Giacchino, Roberto Besana, Giuseppe Ricciardi, Andrea Sterpa, Nicola Altamura, Massimo Andreotti, Giovanni Montrasio, Luigi Macchi, Anna Pavan, Sara Paladini, Alessandro Zanetti, Giovanni Radaelli

**Affiliations:** 1 Department of Paediatrics, Luigi Sacco Hospital, Università di Milano, Milan, Italy; 2 Department of Public Health, Microbiology and Virology, Università di Milano, Milan, Italy; 3 Department of Paediatrics, "Guido Salvini" Hospital, Garbagnate Milanese, Italy; 4 Department of Paediatrics, Ospedale di Desio, Desio, Italy; 5 Department of Paediatrics, Ospedale di Sesto San Giovanni, Sesto San Giovanni, Italy; 6 Department of Paediatrics, Ospedale di Carate Brianza, Carate Brianza, Italy; 7 Department of Paediatrics, Ospedale Provinciale di Saronno, Saronno, Italy; 8 Unit for Prevention, Hygiene and Infectious Diseases of the Health's General Office of Lombardy Region, Milan, Italy; 9 Department of Paediatrics and Unit of Medical Statistics, Università di Milano, San Paolo Hospital, Milan, Italy

## Abstract

**Background:**

Rotavirus is the major cause of acute gastroenteritis and severe dehydrating diarrhea in young children.

**Methods:**

To estimate the proportion of hospital admissions for rotavirus acute gastroenteritis and identify the circulating G and P genotypes among children under five years of age, we conducted a prospective observational study from January to December 2008, recruiting children consecutively admitted to six hospitals in Milan and nearby towns in northern Italy. Typing was done on stool samples by reverse transcriptase polymerase chain reaction amplification.

**Results:**

Of the 521 stool samples from children with acute gastroenteritis, 34.9% (95%CI, 30.8 to 39.2%) were rotavirus-positive. Two thirds (67.6%) were under two years of age, and 13.2% were under six months. The predominant G type was G1 (40.7%), followed by G9 (22.5%), G2 (13.2%), G3 (5.5%), G4 (3.8%) and G10 (1.6%). Twenty-one (11.7%) mixed-G infections were identified: G1+G10 (8.8%); G1+G9 (1.6%); and G2+G10 (1.2%). Only P[8] (67.6%) and P[4] (12.6%) types were P genotyped. The predominant single G/P combination was G1P[8] (39.7%), followed by G9P[8] (25.3%), G2P[4] (14.3%), and G3P[8] (4.1%). All G-mixed types combined with P[8].

**Conclusions:**

These findings show an high prevalence of rotavirus infections among children admitted to hospital for acute gastroenteritis caused by different rotavirus strains circulating in the area studied.

## Background

Rotavirus is the most common cause of acute gastroenteritis and the leading cause of severe dehydrating diarrhea in infants and young children [1]. In Europe, every child is expected to experience episodes of gastroenteritis during the first three years of life [2]. A recent study [3] estimated that there are 3.6 million episodes of rotavirus disease annually among the 23.6 million children under the age of five, living in the European Union, with 231 deaths, more than 87,000 hospitalizations and about 700,000 primary care consultations. In the USA, rotavirus causes an estimated 410,000 medical consultations, more than 200,000 emergency visits, and about 50,000-70,000 hospital admissions per year [4-7].

The admission rate for children with gastroenteritis has not declined in recent years, and may even have risen [6]. The costs of rotavirus gastroenteritis contribute to a critical burden of disease in the pediatric population [8,9]. A recent update of the global burden of rotavirus disease put the median cost of hospital stay in EU-25 Member States at about €1,417 [9].

Rotavirus is composed by 11 double-stranded RNA segments surrounded by three concentric protein layers. The outer capsid consists of VP7 (a glycoprotein) and VP4 (a protease-sensitive protein) which carry independent neutralization and protective antigens. A binary system of rotavirus classification has been established to designate the neutralization specificity of the VP7 and VP4 proteins. The VP7 serotype is known as G and the VP4 serotype as P.

Vaccination has been suggested as a public health strategy to prevent rotavirus infection and reduce the burden of disease [10]. Rotavirus vaccine was recommended for routine use among young children in the USA in 2006. Currently, the World Health Organization recommends including rotavirus vaccines in national immunization programs in regions where the efficacy data suggest a significant public health impact and where appropriate infrastructure and financing resources are available [11]. In Europe, rotavirus gastroenteritis can now be prevented by vaccination with two vaccines (RotaTeq^®^, Merck & Co. Inc, Whitehouse Station, NJ; Rotarix^®^, GlaxoSmithKline Biologicals, Rixensart, Belgium) licensed in 2006. In Italy, no data exists currently about vaccination coverage.

Accurate information on the burden of rotavirus gastroenteritis are crucial to guide recommendations for rotavirus vaccination [12], most epidemiological data have been based on retrospective data. Thus, as the distribution of circulating rotavirus serotypes may vary dynamically, prospective studies are needed to help the health authorities in planning effective immunization strategies, and for industry to look into the possibilities for new up-to-date rotavirus vaccines.

The main aim of this study was to estimate the proportion of rotavirus gastroenteritis and identify the distribution of circulating G and P genotype rotavirus strains among children under five years of age living in the metropolitan area of Milan, northern Italy, and admitted to hospital with acute gastroenteritis. We also examined the relationship between rotavirus genotype and the clinical characteristics of gastroenteritis.

## Methods

This prospective, multicenter, observational study was conducted over a 12-month period, and involved six hospitals in Milan and nearby towns. All children under five years of age at admission and admitted to hospital between 1 January and 31 December 2008 were assessed for eligibility. Inclusion criterion was acute gastroenteritis at enrolment. The exclusion criteria were refusal to give written consent and a previous rotavirus vaccination. Acute gastroenteritis was defined as three or more loose stools per day for less than 14 days at enrolment [13].

The study was conducted in accordance with the Declaration of Helsinki and Good Clinical Practice guidelines. The ethics committee of each hospital reviewed and approved the study protocol. Written informed consent was obtained from the parents or legal guardians at enrolment. No incentive was provided to encourage study participation.

At enrolment, each child was visited by experienced pediatricians (two per center), and underwent a scheduled medical examination including routine assessments.

The pediatricians attended a one-day training course for standardization during the week before starting the study. The pediatricians also interviewed the parents or the legal guardian to collect socio-demographic data, medical history and clinical characteristics of the current episode of acute gastroenteritis.

A stool sample was taken within 18 hours of admission for each child. Approximately 5 g of stool was collected, immediately stored at -20°C, and shipped frozen to the Department of Public Health-Microbiology-Virology of the University of Milan, for analysis. Stool samples were tested for VP6 rotavirus antigen by immuno-chromatographic assay (Orion Diagnostica Inc., Espoo, Finland), according to the manufacturer's instructions. Viral RNA was extracted from 10% fecal suspension using QIAamp MinElute Virus Spin (Qiagen GmBH., Hilden Germany) according to the manufacturer's instructions. The extracted viral RNA was stored at -70°C until testing. Rotavirus RNA was detected by hemi-nested reverse polymerase chain reaction (RT-PCR) employing type-specific primers.

In the first round, a 881 nt fragment of the VP7 region was amplified with generic primers VP7F and VP7R [14]. The G genotypes was subsequently determined on amplicons obtained using a pool of internal primers specific for G1-G4 and G8-G10 rotavirus genotypes in combination with the appropriate reverse consensus primers [14]. Similarly, a 663 nt fragment of the VP4 region was reverse transcribed and amplified using the generic primers VP4F and VP4R, and P genotyping was done using internal primers specific for P[4], P[6], P[8], P[9], P[10] and P[11][15]. Lastly, the amplicons were analyzed by electrophoresis using a 2% agarose gel at 100 V for 60 minutes.

Diarrhea, dehydration status, body rectal temperature, vomiting, and behavioral symptoms (i.e., irritability, lethargy, seizure) were monitored during the hospital stay. Diarrhea was defined in accordance with the International Classification of Diseases (ICD)-9 code 787.91, and vomiting included ICD-9 codes 787.01 and 787.03. Dehydration status was recorded using the accepted reference standard of the 'percentage of volume lost' calculated as the difference between the rehydration weight (post-rehydration body weight) and the acute weight (body weight at presentation) divided by the rehydration weight [16]. Dehydration severity was classified in accordance with the European Society of Pediatric Gastroenterology, Hepatology and Nutrition [17] in three categories, with body weight loss < 5%, 5-10%, >10%. The 20-point Ruuska-Vesikari scale [18] was used as a further overall indicator of the severity of acute gastroenteritis. This scale assigns points according to duration and intensity of diarrhea, vomiting, body temperature, behavioral symptoms, and the treatment given. Ruuska-Vesikari defines severity of disease as: mild, score ≤6; moderate, score 7-10; severe, score ≥11.

### Statistical analysis

The Kolmogorov-Smirnov test was used to assess normality of distribution of the variables. All variables except age were not symmetrically distributed (*P *< 0.05). Descriptive data for age are reported as mean and standard deviation (SD). For the other variables we report the median (range) or number of observations (percentage). The proportion of rotavirus gastroenteritis among all the hospital admissions for acute gastroenteritis was calculated with the exact binomial 95% confidence interval (CI). Comparisons of age for the rotavirus genotypes were tested by one-way ANOVA. Comparisons of rotavirus genotypes for non-symmetric variables were done the Kruskal-Wallis test, followed by the non-parametric multiple comparisons Tukey's test. A *P-*value < 0.05 was considered to indicate statistical significance (two-tailed test). All statistical analyses were done using the SPSS software, version 15.0 (SPSS Inc., Chicago, IL).

## Results

A total of 638 children aged less than 5 years were admitted with acute gastroenteritis (374 boys, 264 girls; mean [±SD] age 1.5 ±1.3 years). Stool samples were tested to 521 (81.7%) children: 182 (110 boys, 72 girls; mean age 1.7 ± 1.1 years) were positive for rotavirus, namely 34.9% (95%CI, 30.8-39.2%). There were 123 cases of rotavirus gastroenteritis in children under two years old (67.6%): 24 (13.2%) in infants aged < 6 months; 37 (20.3%) in infants aged 6 to < 12 months; and 62 (34.1%) in infants aged 1 to < 2 years.

Severe acute gastroenteritis occurred in 126 (69.2%) rotavirus-positive children and in 140 (41.4%) rotavirus-negative children (*P *< 0.0001), with no difference between groups in duration of hospital stay (median and range 4 (1-10) vs. 4 (1-9) days) (*P *= 0.718).

**Figure 1:**
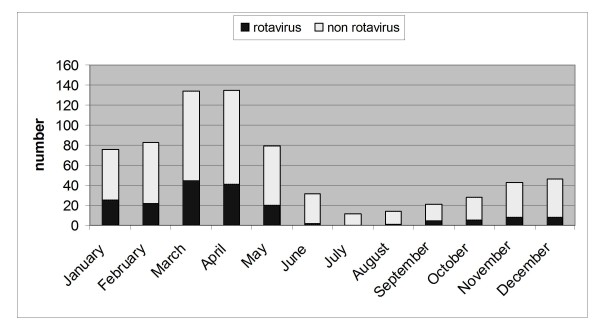
**Number of admissions for acute rotavirus gastroenteritis by month, in the area of Milan, northern Italy (%)**.

**Figure 2:**
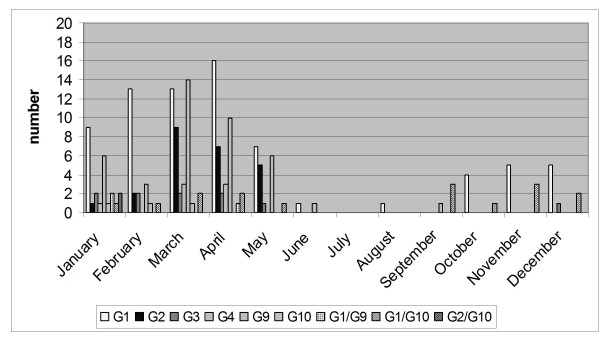
**Number of typed rotavirus G genotypes by month, in the area of Milan, northern Italy**.

Admissions for rotavirus gastroenteritis showed seasonal differences (*P *< 0.001), with more cases in spring (85; 46.7%) and winter (74; 40.7%) than autumn (19; 10.4%) and summer (4; 2.2%) (Figure 1). All G types were observed both in winter and spring. The four cases occurring in summer were one single G1, one single G9 and two G1+G10. Among cases occurring in autumn 18 were G1 (12 G1 alone, and 6 G1+G10) and one G3. Figure 2 shows the distribution of the typed rotavirus G genotypes over the year.

Among the 182 rotavirus-positive cases, 180 (98.9%) were identified for G genotype and 146 (80.2%) for P genotype (Table 1). One case (girl, age 3 months) was neither G nor P genotyped. Single G types were observed in 159 (87.4%) cases, mixed-G types in 21 (11.5%). G1 (74, 40.7%) was the predominant single rotavirus G type, followed by G9 (41, 22.5%), G2 (24, 13.2%), G3 (10, 5.5%), G4 (7, 3.8%) and G10 (3, 1.6%). Mixed-G types were identified as G1+G10 (16, 8.9%), G1+G9 (3, 1.6%) and G2+G10 (2, 1.1%). Only P[8] (123, 67.6%) and P[4] (23, 12.6%) types were found. In 146 cases genotyped for both G and P, the predominant combination was G1P[8] (58, 39.7%), followed by G9P[8] (37, 25.3%) and G2P[4] (21, 14.3%). Each mixed-G type combined with P[8].

**Table 1:** Distribution of rotavirus strains circulating among children hospitalized with acute gastroenteritis in the area of Milan, Northern Italy.

	P genotype			
		
G genotype(s)	P[8]	P[4]	P-negative	Total
G1	58 (47.2)	0	16 (44.4)	74 (40.7)
G2	0	21 (91.3)	3 (8.3)	24 (13.2)
G3	6 (4.9)	2 (8.7)	2 (5.6)	10 (5.5)
G4	6 (4.9)	0	1 (2.8)	7 (3.8)
G9	37 (30.1)	0	4 (11.1)	41 (22.5)
G10	0	0	3 (8.3)	3 (1.6)
G1+G9	3 (2.4)	0	0	3 (1.6)
G1+G10	11 (8.9)	0	5 (13.9)	16 (8.9)
G2+G10	1 (0.8)	0	1 (2.8)	2 (1.1)
G-negative	1 (0.8)	0	1 (2.8)	2 (1.1)
Total	123 (100)	23 (100)	36 (100)	182 (100)

Data are numbers (percentage) of children

The rotavirus gastroenteritis was rated as mild, moderate or severe in 12 (6.6%), 44 (24.2%) and 126 (69.2%) children, respectively (Ruuska-Vesikari scale). Dehydration was < 5%, 5-10%, > 10%, in 158 (86.7%), 22 (12.2%), and 2 (1.1%) children (one girl aged 20 months with G3P[4], and one girl aged 47 months with G2P[4]). The median (range) duration of diarrhea was 5 (1-11) days. The median (range) maximum rectal temperature was 38.8°C (36.6-40.4). Vomiting occurred in 164 (90.1%) children and 133 (73.1%) presented behavioral symptoms. Oral rehydration was required for 129 (70.9%) children, intravenous rehydration for 138 (75.8%).

Table 2 summarizes the main clinical characteristics of the rotavirus gastroenteritis, according to G and P genotypes. Duration (median, range) of diarrhea was shorter in G3 (3, 2-4 days) than in G1 (5, 1-10 days) and G9 (5, 2-10 days) (both *P *< 0.05). Duration of diarrhea was associated with severity of gastroenteritis (Spearman correlation coefficient, r = 0.423, *P *< 0.0001), percentage of dehydration (r = 0.168, *P *= 0.023) and duration of hospital stay (r = 0.554, *P *< 0.0001), and was longer in younger children (r = -0.155, *P *< 0.0001). Maximum rectal temperature was lower in G1+G10 than in G3 (39.6°C, 36.8-40.4) (*P *< 0.01). Dehydration differed among G subtypes (*P *< 0.001) and was higher in G1 (1.8, 0-10%) and G3 (1.9, 0-10.9%) than G9 (0, 0-8.2%) (*P *< 0.01) but the differences between G genotypes were not significant for severity of dehydration (*P *> 0.287). Similarly, the differences between G genotypes were not significant for Ruuska-Vesikari scores (*P *= 0.610), number of days with vomiting (*P *= 0.328), or behavioral symptoms (*P *= 0.417). There were no significant differences between P genotypes for any of the clinical characteristics (minimum *P *= 0.09 for duration of diarrhea).

**Table 2:** Clinical characteristics of rotavirus-positive children hospitalized with rotavirus acute gastroenteritis by G and P genotypes.

Genotype	Ruuska-Vesikari scale	Dehydration (%)	Diarrhea (no. of days)	Maximum rectal temperature (°C)	Vomiting (no. of days)	Behavioral symptoms
G1 (n = 74)	13 (5-19)	1.8 (0-10)	5 (1-10)	38.7 (36.6-40)	2 (0-7)	55 (74.3)
G2 (n = 24)	13 (8-19)	0 (0-13.2)	4.5 (2-8)	38.7 (36.8-40.0)	2 (0-4)	18 (75.0)
G3 (n = 10)	13 (9-15)	1.9 (0-10.9)	3 (2-4)	39.6 (36.8-40.4)	1 (0-4)	7 (70.0)
G4 (n = 7)	14 (7-17)	0 (0-6.0)	5 (2-5)	39.0 (38.3-39.5)	1 (1-2)	5 (71.4)
G9 (n = 41)	13 (5-17)	0 (0-8.2)	5 (2-10)	38.8 (36.8-40.0)	2 (0-6)	29 (70.7)
G10 (n = 3)	14 (4-15)	2.6 (2.5-3.9)	4 (2-11)	39.2 (39.0-39.5)	2 (0-2)	2 (66.7)
G1+G9 (n = 3)	13 (11-13)	2.2 (1.1-3.5)	4 (4-5)	39.0 (38.7-39.0)	2 (1-6)	2 (66.7)
G1+G10 (n = 16)	12 (7-16)	0 (0-5.6)	5 (4-7)	38.0 (36.7-39.2)	2 (0-4)	11 (68.7)
G2+G10 (n = 2)	13.5 (11-16)	4.7 (2.6-6.8)	6 (3-9)	39.7 (39.5-39.9)	1 (1-1)	2 (100.0)
Nontypeable for G (n = 2)	11.5 (10-13)	0 (0-0)	5.5 (4-7)	37.7 (37.0-38.4)	2.5 (1-4)	1 (50.0)
P 8 (n = 123)	13 (5-19)	1.3 (0-10)	5 (1-9)	38.8 (36.7-40.0)	2 (0-6)	89 (72.4)
P 4 (n = 23)	13 (8-19)	0 (0-13.2)	4.5 (2-8)	38.7 (36.8-40.4)	2 (0-3)	16 (69.6)
Nontypeable for P (n = 36)	13 (4-19)	0.7 (0-10)	5 (2-11)	38.8 (36.6-40-0)	2 (0-7)	21 (58.3)
Whole sample	13 (4-19)	0.75 (0-13.2)	5 (1-11)	38.8 (36.6-40.4)	2 (0-7)	133 (73.1)

Median (range) or number (percentage) of children

Mean age and sex distribution did not differ for G or P genotypes (minimum *P *> 0.167). Duration of hospital stay did not differ among G types (*P *= 0.250), ranging from a median (range) of 3 (3-5) days with G10 to 7 (4-10) days with G2+G10. Median (range) hospital stay was respectively 5 (1-9) days and 4.5 (2-8) days in the P[8] and P[4] subgroups, and did not differ with the P non-typeable (36) cases (median 5, range 2-11 days) (*P *> 685).

## Discussion

Continuous monitoring of rotavirus genotype distribution would be valuable to show up the diversity and changes in the circulating strains that may be more important than ever since the introduction of rotavirus vaccination. Updating epidemiological data and extending knowledge worldwide might prove useful for designing up-to-date vaccine-based prevention strategies.

The present study is the first to provide information on rotavirus distribution in northern Italy. It prospectively investigated the rotavirus genotype distribution among children under five years of age living in the metropolitan area of Milan admitted to hospital for acute gastroenteritis. It also looked into possible relations between the clinical characteristics of disease and the rotavirus genotype.

The findings confirmed that rotavirus is a major cause of hospitalization for acute gastroenteritis among children under five years of age, accounting for 34.9% of cases. In Europe, it was estimated that in the period 2004-2005 about 10.4% to 36.0% of children aged five years or younger and admitted with acute gastroenteritis had disease caused by rotavirus [19]. More recently, in a prospective study conducted in five European Union countries (France, Germany, Italy, Spain, and the United Kingdom) in children aged < 5 years, rotavirus accounted for 56.2% of all hospitalized community-acquired acute gastroenteritis cases, ranging from 33.2% in Italy to 64.4% in France [20]. There also appears to be a substantial burden of rotavirus hospitalization among young children in the United States, [6,7,21,22] with rotavirus accounting for about 18.4% of annual hospital admissions for acute gastroenteritis among children < 0.5 years of age [6].

In our study, most admissions for acute rotavirus gastroenteritis were infants, with 13.2% aged less than six months, and two among the very youngest (less than two months old). These figures are slightly lower than recent European surveillance data showing that rotavirus gastroenteritis ranged from 56.7% to 74.2% of cases of acute gastroenteritis in infants aged 6 to 23 months, and from 18.1% to 31.9% in infants aged < 6 months [23].

The present study found a clear seasonal pattern of acute rotavirus gastroenteritis that peaked in winter and early spring, with only 2.2% of cases in summer. It is conceivable that when there is a seasonal pattern children may become infected at later ages, because they have not been continuously exposed to the virus [24]. Indeed, seasonality of rotavirus has been observed in different parts of the world; thus, it would be better for future studies to analyse samples from a "season" rather than a "calendar year".

G1 was the predominant rotavirus G type, found in 51.1% of cases (40.7% alone; 10.4% in mixtures), and collectively with G9 (22.5% alone; 1.6% in mixtures) and G2 (13.2% alone; 1.1% in mixtures) it accounted for more than 90% of the rotavirus strains identified. In an extensive review published in 2005 Santos and Hoshino [25] merged a total of 45571 cases collected worldwide from 124 studies, and estimated that G1, G2, G3 and G4 covered about 90% of rotavirus G types. In the present study we found a higher proportion of G9 than the 7.4% reported by De Donno et al. [26], 2.2%-11.1% by Tcheremenskaia [27] (except in Bulgaria where the rate of G9 was 36.6%), and 1.3-9.6% by Santos et al. [25]; however, our data compares well with a recent European study [20] that found G9 (31.2%) was the second most prevalent circulating rotavirus G type after G1 (40.3%). In the present study the prevalence of G3 was comparable with previous reports [20,25] while circulation of G4 was lower.

Geographic and temporal differences in rotavirus genotype distribution may explain at least some of these differences, as has been pointed out in several studies worldwide [20,25,28,29]. Recognition of G10 alone confirms the presence of this rotavirus genotype in Italy and in other European countries, as reported recently [26,27]. As a matter of fact, the prevalence (10%) of G10 mixed-G types is higher than previously reported. However it would be important to underline that 83.3% (15/18) of patients with such genotypes were admitted to the same hospital in a restricted period of time (September-December).

Epidemiological surveys showed that this genotype may be detected in increasing proportions of infants and young children with acute gastroenteritis in parts of India [30], Brazil [31], Paraguay [32], and Thailand [33]. Although interspecies transmission of animal rotavirus to humans appears to be a very rare event, the introduction of certain animal rotavirus genes into human rotaviruses through genetic reassortment appears to be common, as in the case of G10 [25].

Lastly, in the present study the proportion of G-mixed types was in the found previously, ranging from about 1.6% to 12.7%.

Among P genotypes, P[8] and P[4] accounted for all P typed cases. The predominant combinations were G1P[8] (44.1%), G9P[8] (35.6%), G4P[8] (5.1%) and G3P[8] (3.4%). A similar distribution was found in Europe recently by Forster et al. [20], with G1P[8], 40.3%; G9P[8], 31.2%; G4P[8], 13.5%; and G3P[8], 7.1%). Other reports show G1P[8], G9P[8], G4P[8], G3P[8], and G2P[4] as collectively responsible for more than 90% of the rotavirus infections in Western countries, about 70% in South America and Asia, and 50% in Africa [25]. The importance of G9P[8] in Italy was pointed out by De Donno et al. [26]. This type was relatively common in South America (15%), Asia (22% in Bangladesh between 2000 and 2006 [34], 8.5% in India between 2005-2007 [35], 33.8% and 54.8% in 2001 and 2002 in Taiwan [36]) and Africa (15% in Nigeria in 2006 [37]), and possibly less frequent in the United States where a recent ten-year pre-vaccine surveillance (1996 to 2005) estimated the overall rate at 3.6%, but it has now really risen to 31% in Europe [20].

Some caution is needed, however, in interpreting the findings of the present study concerning the P genotype because of the fairly large proportion (19.8%) of P non-typeable samples. While this proportion is lower than in some other studies (e.g. 38.6% in [32]), Forster et al. [20] managed to genotype all samples. Since rotaviruses genetically mutate (sequential point mutations, genetic reassortment, genomic rearrangement or intragenic recombination) [38,39], it is to be expected that sometimes today's RT-PCR methodologies are unable to identify all types. Unusual G/P combinations have been described worldwide and may amount globally to about 5% of all rotavirus types. The appearance of new strains may reflect naturally occurring reassortants among various human rotavirus genotypes but might also involve reassortants between human and animal strains and/or result from direct transmission from animals to humans [40].

As regards how the rotavirus genotype was related to the clinical features of acute gastroenteritis, we found no statistically significant or clinically relevant differences as a whole between different G or P genotypes in the severity of gastroenteritis, rated using the Ruuska-Versikari scale, or the severity of dehydration, but diarrhea lasted about two days less with G3 than either G alone or mixed types. However, these conclusions are based on some very small subgroups of rotavirus types, and this must be borne in mind, together with the wide coefficient of variation of the clinical characteristics of rotavirus gastroenteritis. Larger, adequately powered longitudinal studies are needed to examine any possible relationships between the clinical profile of rotavirus acute gastroenteritis and G and P types.

## Conclusions

In conclusion, the present study confirms the current burden of rotavirus gastroenteritis in younger children, especially small infants, and shows up the diversity of rotavirus strains circulating in Milan and neighboring areas Continuous prospective monitoring of circulating strains may be desirable to detect any change in the distribution of rotavirus strains promptly and to assess the effectiveness of active immunization programs.

## Competing interests

The authors declare that they have no competing interests.

## Authors' contributions

GZ, FM, DD, RB, CM, RG and RB made substantial contributions to the study conception and design, data acquisition, analysis and interpretation. LR, GR and AS helped draft the manuscript or revise it critically for important intellectual content. NA, MA, GM, LM, AP, AZ and GR gave final approval of the version to be published.

All authors have given final approval of the version to be published

## Pre-publication history

The pre-publication history for this paper can be accessed here:

http://www.biomedcentral.com/1471-2334/10/218/prepub

## References

[B1] Parashar UD Hummelman EG Bresee JS Miller MA Glass RI Global illness and deaths caused by rotavirus disease in children Emerg Infect Dis 2003 9 565 572 12737740 10.3201/eid0905.020562 PMC2972763

[B2] Clark B McKendrick M A review of viral gastroenteritis Curr Opin Infect Dis 2004 17 461 469 10.1097/00001432-200410000-00011 15353966

[B3] Soriano-Gabarrò M Mzrukowic J Vesikari T Burden of Rotavirus disease in European Union countries Pediatr Infect Dis J 2006 25 Suppl 1 S7 S11 16397431 10.1097/01.inf.0000197622.98559.01

[B4] Parashar UD Holman RC Clarke MJ Bresee JS Glass RI Hospitalizations associated with rotavirus diarrhoea in the United States 1993 through 1995: surveillance based on the new ICD-9-CM rotavirus-specific diagnostic code J Infect Dis 1997 177 13 17 10.1086/513808 9419164

[B5] Tucker AW Haddix AC Bresee JS Holman RC Parashar UD Glass RI Cost-effectiveness analysis of a rotavirus immunization program for the United States JAMA 1998 279 1371 1376 10.1001/jama.279.17.1371 9582045

[B6] Charles MD Holman RC Curns AT Parashar UD Glass RI Breese JS Hospitalizations associated with rotavirus gastroenteritis in the United States 1993-2002 Pediatr Infect Dis J 2006 25 489 493 10.1097/01.inf.0000215234.91997.21 16732145

[B7] Malek MA Curns AT Holman RC Fischer TK Bresee JS Glass RI Steiner CA Parashar UD Diarrhea-and rotavirus-associated hospitalizations among children less than 5 years of age: United States 1997 and 2000 Pediatrics 2006 117 1887 1892 10.1542/peds.2005-2351 16740827

[B8] Lorgelly PK Joshi D Iturriza Gómara M Flood C Hughes CA Dalrymple J Gray J Mugford M Infantile gastroenteritis in the community: a cost-of-illness study Epidemiol Infect 2008 136 34 43 17338837 10.1017/S0950268807008163 PMC2870764

[B9] Pediatric ROTavirus European CommitTee (PROTEC) The paediatric burden of rotavirus disease in Europe Epidemiol Infect 2006 134 908 916 10.1017/S0950268806006091 16650331 PMC2870494

[B10] Glass RI Parashar UD Bresee JS Turcios R Fischer TK Widdowson MA Jiang B Gentsch JR Rotavirus vaccines: current prospects and future challenges Lancet 2006 368 323 332 10.1016/S0140-6736(06)68815-6 16860702

[B11] World Health Organization Rotavirus vaccines. WHO position paper Wkly Epidemiol Rec 2007 82 285 29617691162

[B12] Van Damme P Van der Wielen M Ansaldi F Desgrandchamps D Domingo JD Sanchez FG Gray J Haditsch M Johansen K Lorgelly P Lorrot M Parez N Reschke V Rose M Rotavirus vaccines: considerations for successful implementation in Europe Lancet Infect Dis 2006 6 805 812 10.1016/S1473-3099(06)70657-0 17123900

[B13] Riordan FA Quigley T Estimating hospital admissions due to rotavirus gastroenteritis from hospital episode statistics J Infect 2004 49 13 16 10.1016/j.jinf.2004.02.006 15194242

[B14] Iturriza-Gómara M Kang G Gray J Rotavirus genotyping: keeping up with an evolving population of human rotaviruses J Clin Virol 2004 31 259 265 10.1016/j.jcv.2004.04.009 15494266

[B15] Simmonds MK Armah G Asmah R Banerjee I Damanka S Esona M Gentsch JR Gray JJ Kirkwood C Page N Iturriza-Gómara M New oligonucleotide primers for P-typing of rotavirus strains: Strategies for typing previously untypeable strains J Clin Virol 2008 42 368 373 10.1016/j.jcv.2008.02.011 18378188

[B16] National Collaborating Centre for Women's and Children's Health Diarrhoea and vomiting caused by gastroenteritis diagnosis assessment and management in children younger than 5 years 2009 London: RCOG Press http://www.nice.org.uk/nicemedia/pdf/CG84FullGuideline.pdf Available at.Last accessed 17 11 2009 22132432

[B17] Sandhu BK European Society of Pediatric Gastroenterology Hepatology and Nutrition Working Group on Acute Diarrhoea Practical guidelines for the management of gastroenteritis in children J Pediatr Gastroenterol Nutr 2001 33 Suppl 2 S36 S39 10.1097/00005176-200110002-00007 11698784

[B18] Ruuska T Vesikari T Rotavirus disease in Finnish children: use of numerical scores for clinical severity of diarrhoeal episodes Scand J Infec Dis 1990 22 259 267 10.3109/00365549009027046 2371542

[B19] Giaquinto C Van Damme P Huet F Gothefors L Maxwell M Todd P da Dalt L REVEAL Study Group. Clinical consequences of rotavirus acute gastroenteritis in Europe 2004-2005: the REVEAL study J Infect Dis 2007 195 Suppl 1 S26 S35 10.1086/516717 17387649

[B20] Forster J Guarino A Parez N Moraga F Román E Mory O Tozzi AE de Aguileta AL Wahn U Graham C Berner R Ninan T Barberousse C Meyer N Soriano-Gabarró M the Rotavirus Study Group Hospital-based surveillance to estimate the burden of rotavirus gastroenteritis among European children younger than 5 years of age Pediatrics 2009 123 e393 e400 10.1542/peds.2008-2088 19254975

[B21] Fischer TK Viboud C Parashar U Malek M Steiner C Glass R Simonsen L Hospitalizations and deaths from diarrhea and rotavirus among children < 5 years of age in the United States 1993-2003 J Infect Dis 2007 195 1117 1125 10.1086/512863 17357047

[B22] Patel MM Tate JE Selvarangan R Daskalaki I Jackson MA Curns AT Coffin S Watson B Hodinka R Glass RI Parashar UD Routine laboratory testing data for surveillance of rotavirus hospitalizations to evaluate the impact of vaccination Pediatr Infect Dis J 2007 26 914 919 10.1097/INF.0b013e31812e52fd 17901797

[B23] Giaquinto C Van Damme P Huet F Gothefors L Van der Wielen M REVEAL Study Group Multicenter prospective study of the burden of rotavirus acute gastroenteritis in Europe 2004-2005: the REVEAL Study J Infect Dis 2007 195 S4 S16 10.1086/516714 17387650

[B24] Pérez-Schael I González R Fernández R Alfonzo E Inaty D Boher Y Sarmiento L Epidemiological features of rotavirus infection in Caracas Venezuela: implications for rotavirus immunization programs J Med Virol 1999 59 520 526 10.1002/(SICI)1096-9071(199912)59:4<520::AID-JMV16>3.0.CO;2-7 10534736

[B25] Santos N Hoshino Y Global distribution of rotavirus serotypes/genotypes and its implication for the development and implementation of an effective rotavirus vaccine Rev Med Virol 2005 15 29 56 10.1002/rmv.448 15484186

[B26] De Donno A Grassi T Bagordo F Idolo A Cavallaro A Gabutti G Collaborative Group for the surveillance of Rotavirus Infection. Emergence of unusual human rotavirus strains in Salento Italy, during 2006-2007 BMC Infect Dis 2009 9 43 10.1186/1471-2334-9-43 19368717 PMC2676288

[B27] Tcheremenskaia O Marucci G De Petris S Ruggeri FM Dovecar D Sternak SL Matyasova I Dhimolea MK Mladenova Z Fiore L Rotavirus Study Group. Molecular epidemiology of rotavirus in Central and Southeastern Europe J Clin Microbiol 2007 45 2197 2204 10.1128/JCM.00484-07 17507520 PMC1933006

[B28] Desselberger U Wolleswinkel-van den Bosch J Mrukowicz J Rodrigo C Giaquinto C Vesikari T Rotavirus types in Europe and their significance for vaccination Pediatr Infect Dis J 2006 25 Suppl 1 S30 S41 16397427 10.1097/01.inf.0000197707.70835.f3

[B29] Gentsch JR Hull JJ Teel EN Kerin TK Freeman MM Esona MD Griffin DD Bielfelt-Krall BP Banyai K Jiang B Cortese MM Glass RI Parashar UD collaborating laboratories of the National Rotavirus Strain Surveillance System G and P types of circulating rotavirus strains in the United States during 1996-2005: nine years of prevaccine data J Infect Dis 2009 200 Suppl 1 S99 S105 10.1086/605038 19817622

[B30] Iturriza Gómara M Kang G Mammen A Jana AK Abraham M Desselberger U Brown D Gray J Characterization of G10P[11] rotaviruses causing acute gastroenteritis in neonates and infants in Vellore India J Clin Microbiol 2004 42 2541 2547 10.1128/JCM.42.6.2541-2547.2004 15184432 PMC427862

[B31] Araújo IT Ferreira MS Fialho AM Assis RM Cruz CM Rocha M Leite JP Rotavirus genotypes P[4]G9, P[6]G9, and P[8]G9 in hospitalized children with acute gastroenteritis in Rio de Janeiro Brazil J Clin Microbiol 2001 39 1999 2001 10.1128/JCM.39.5.1999-2001.2001 11326034 PMC88069

[B32] Coluchi N Munford V Manzur J Vazquez C Escobar M Weber E Mármol P Rácz ML Detection subgroup specificity and genotype diversity of rotavirus strains in children with acute diarrhea in Paraguay J Clin Microbiol 2002 40 1709 1714 10.1128/JCM.40.5.1709-1714.2002 11980947 PMC130658

[B33] Urasawa S Hasegawa A Urasawa T Taniguchi K Wakasugi F Suzuki H Inouye S Pongprot B Supawadee J Suprasert Sl Antigenic and genetic analyses of human rotavirus in Chiang Mai Thailand: evidence for a close relationship between human and animal rotaviruses J Infect Dis 1992 166 227 234 1378870 10.1093/infdis/166.2.227

[B34] Zaman K Yunus M Faruque AS El Arifeen S Hossain I Azim T Rahman M Podder G Roy E Luby S Sack DA Surveillance of rotavirus in a rural diarrhoea treatment centre in Bangladesh 2000-2006 Vaccine 2009 27 5 F31 34 10.1016/j.vaccine.2009.08.063 19931715

[B35] Kang G Arora R Chitambar SD Deshpande J Gupte MD Kulkarni M Naik TN Mukherji D Venkatasubramaniam S Gentsch JR Glass RI Parashar UD Indian Rotavirus Strain Surveillance Network.Multicenter hospital-based surveillance of rotavirus disease and strains among Indian children aged < 5 years J Infect Dis 2009 200 1 S147 153 10.1086/605031 19817593

[B36] Lin YP Chang SY Kao CL Huang LM Chung MY Yang JY Chen HY Taniguchi K Tsai KS Lee CN Molecular epidemiology of G9 rotaviruses in Taiwan between 2000 and 2002 J Clin Microbiol 2006 44 3686 3694 10.1128/JCM.02107-05 17021098 PMC1594809

[B37] Steele AD Nimzing L Peenze I De Beer MC Geyer A Angyo I Gomwalk NE Circulation of the novel G9 and G8 rotavirus strains in Nigeria in 1998/1999 Journal of medical virology 2002 67 608 612 10.1002/jmv.10146 12116012

[B38] Suzuki Y Gojobori T Nakagomi O Intragenic recombination in rotaviruses FEBS Lett 1998 427 183 187 10.1016/S0014-5793(98)00415-3 9607308

[B39] Desselberger U Genome rearrangements of rotavirus Adv Virus Res 1996 49 69 95 full_text 10.1016/s0065-3527(08)60070-6 8824698

[B40] Iturriza-Go´mara M Isherwood B Desselberger U Gray J Reassortment in vivo: driving force for diversity of human rotavirus strains isolated in the United Kingdom between 1995 and 1999 J Virol 2001 75 3696 3705 10.1128/JVI.75.8.3696-3705.2001 11264359 PMC114861

